# Diagnostic and public health investigation of *Mycobacterium tuberculosis* infection in a dog in Ontario, Canada

**DOI:** 10.1177/10406387221074706

**Published:** 2022-01-25

**Authors:** Luke A. J. Haydock, Anthony C. G. Abrams-Ogg, J. Scott Weese, Michael R. Goldstein, Amy B. Clifford, Adrian Sebastian, Elizabeth H. Rea, Frances B. Jamieson, Carla Duncan, Olga Andrievskaia, Mirjana Savic, Durda Slavic, Robert A. Foster, Christopher J. Greenwood, Tamara L. MacDonald, Jacqueline E. Scott, Andrea Sanchez

**Affiliations:** Department of Pathobiology, University of Guelph, Guelph, Ontario, Canada; Health Sciences Centre, University of Guelph, Guelph, Ontario, Canada; Ontario Veterinary College, and Centre for Public Health and Zoonoses, University of Guelph, Guelph, Ontario, Canada; Central Toronto Veterinary Referral Clinic, Toronto, Ontario, Canada; Toronto Public Health, Toronto, Ontario, Canada; Toronto Public Health, Toronto, Ontario, Canada; Toronto Public Health, Toronto, Ontario, Canada; Public Health Ontario, Toronto, Ontario, Canada; Public Health Ontario, Toronto, Ontario, Canada; Ottawa Laboratory Fallowfield, Canadian Food Inspection Agency, Ottawa, Ontario, Canada; Ottawa Laboratory Fallowfield, Canadian Food Inspection Agency, Ottawa, Ontario, Canada; Animal Health Laboratory, University of Guelph, Guelph, Ontario, Canada, University of Guelph, Guelph, Ontario, Canada; Department of Pathobiology, University of Guelph, Guelph, Ontario, Canada, University of Guelph, Guelph, Ontario, Canada; Health Sciences Centre, University of Guelph, Guelph, Ontario, Canada, University of Guelph, Guelph, Ontario, Canada; Health Sciences Centre, University of Guelph, Guelph, Ontario, Canada, University of Guelph, Guelph, Ontario, Canada; Health Sciences Centre, University of Guelph, Guelph, Ontario, Canada, University of Guelph, Guelph, Ontario, Canada; Health Sciences Centre, University of Guelph, Guelph, Ontario, Canada, University of Guelph, Guelph, Ontario, Canada

**Keywords:** canine, *Mycobacterium tuberculosis*, public health

## Abstract

A 4-y-old, female mixed-breed dog was presented to the Ontario Veterinary College for further evaluation of multiple pulmonary and hepatic masses, intrathoracic lymphadenitis, and recent development of a pyogranulomatous pleural effusion. Along with other comprehensive tests, a thoracic lymph node biopsy was performed, and *Mycobacterium tuberculosis* complex infection was confirmed by real-time PCR. The dog’s condition declined post-operatively, and euthanasia was elected. Postmortem examination confirmed severe granulomatous pneumonia, hepatitis, intrathoracic and intraabdominal lymphadenitis, omentitis, and nephritis. Line-probe assays performed on samples collected postmortem confirmed the species as *M. tuberculosis*. 24-loci MIRU-VNTR genotyping, spoligotyping, and whole-genome sequencing revealed relations to known human isolates, but no epidemiologic link to these cases was investigated. Given the concern for potential human exposure during this animal’s disease course, a public health investigation was initiated; 45 individuals were tested for *M. tuberculosis* exposure, and no subsequent human infections related to this animal were identified. Our case highlights the need for more readily available, minimally invasive testing for the diagnosis of canine mycobacteriosis, and highlights the ability of canid species to act as potential contributors to the epidemiology of *M. tuberculosis* infections.

A 4-y-old, spayed female, mixed-breed dog was presented to the Health Sciences Centre, Ontario Veterinary College (OVC; Guelph, Ontario, Canada) in June 2019 for further evaluation of previously diagnosed pulmonary and hepatic masses, intrathoracic granulomatous lymphadenitis, and recent pyogranulomatous pleural effusion. The dog had been adopted from a Quebec-based rescue agency and had spent the prior 18–24 mo living in Toronto, Ontario. Diagnostic imaging prior to referral included thoracic radiographs, computed tomography (CT), and abdominal ultrasound examination. In addition to imaging, previous testing included complete blood counts, serum biochemistry profiles, cytology and culture of bronchoalveolar lavage fluid, cytology and bacterial and fungal cultures of pleural effusion, endoscopic gastric and intestinal biopsies, and fecal Baermann and ELISA for parasitic antigens—the results of which were all largely unremarkable save for mild neutrophilia, thrombocytosis, and mild increases in liver enzyme activities (ALT = 236 IU/L, RI: 10–109 IU/L; ALP = 439 IU/L, RI: 1–114 IU/L]).

*Bartonella* PCR and culture (Galaxy Diagnostics), enzyme immunoassay for *Blastomyces* antigen (MiraVista Diagnostics), and testing for *Anaplasma* (Idexx 4Dx test), *Babesia* (Vector Borne Disease Diagnostic Laboratory, North Carolina State University [VBDL-NCSU]), *Ehrlichia* (Idexx 4Dx test and VBDL-NCSU), *Hepatozoon* (blood smear cytology), *Mycoplasma* (culture performed by Animal Health Laboratory, Guelph), *Rickettsia rickettsii* (Idexx 4Dx test and VBDL-NCSU), and *Leishmania* (cytology and/or histology) also all returned negative results. Additionally, stains performed on tissues and fluids collected thus far had failed to demonstrate the presence of any acid-fast organisms.

Referral was then pursued following a 2-wk period of progressive worsening of pleural effusion, onset of chronic vomiting and lethargy, appearance of a marked inflammatory leukogram, and marked worsening of liver enzyme activities (ALT = 767 IU/L; ALP = 1,960 IU/L; GGT = 63 IU/L, RI: 0–25 IU/L).

Upon initial presentation at OVC, the patient was moderately tachycardic, tachypneic, and pyrexic (39.8°C). Thoracic auscultation was muffled with absent lung sounds ventrally, consistent with severe bilateral pleural effusion, which was confirmed by thoracic ultrasonography. Complete blood count and biochemistry revealed findings similar to those detailed prior to presentation. Following sedation, a thoracostomy tube was placed in the right lateral thorax and ~1,150 mL of serosanguineous fluid was extracted. Aerobic and anaerobic cultures of pleural fluid were negative; cytology indicated mild-to-moderate pyogranulomatous inflammation. Ultrasound-guided aspirates of the previously identified liver nodules were performed; cytology of these samples indicated hepatocellular necrosis; mixed, predominantly suppurative, inflammation; and vacuolar hepatopathy consistent with lipid accumulation and cholestasis. Smears of scant abdominal fluid were also submitted and revealed mixed, predominantly suppurative, inflammation.

Thoracic radiographs, abdominal ultrasound examination, and a thoracic CT scan and lymphangiogram were then performed. Myriad findings included marked mineralized intrathoracic lymphadenopathy; a mineral-dense left caudal pulmonary mass; patchy mineral-dense infiltrative pulmonary disease; bilateral pleural free fluid; multifocal hepatic mineralization with mineral-dense hepatic masses; hepatic, splenic, and para-aortic lymphadenopathy; central and multifocal renal mineralization; and atelectasis in the right cranial lung lobe.

Serology for antinuclear antibody, perinuclear antineutrophil cytoplasmic antibodies, and a fungal serology panel testing for antibodies against *Aspergillus*, *Blastomyces*, *Coccidioides*, *Histoplasma*, and *Cryptococcus* were then submitted, and all ultimately returned negative results. Given previous inconclusive results despite extensive work-up, exploratory thoracotomy was then performed with the intent to obtain biopsies of suspected primary lung lesions identified on CT. Hemorrhage encountered during surgery precluded dissection to the lung lesions, and necessitated emergency blood transfusion, during which time a staff member sustained a needlestick injury. At the time of surgery, mediastinal lymph nodes were sampled for histopathology, impression smear cytology, and flow cytometry to identify clonal lymphocyte populations. In addition, aerobic and anaerobic bacterial culture, fungal culture and wet mount, *Sporothrix schenckii* real-time PCR (rtPCR), *Bartonella* ePCR (digital PCR following enrichment in *Bartonella* alpha-proteobacteria growth medium), and *M. tuberculosis* complex rtPCR were performed on the lymph nodes.

Histopathology of mediastinal lymph nodes revealed effacement by coalescing, necrotic granulomas consisting of an almost pure population of macrophages (termed “naked” granulomas). Initial examination with Ziehl–Neelsen, Fite acid-fast, Gram, periodic acid–Schiff, and Warthin–Starry stains did not highlight any causative microorganisms. Lymph node impression smears were indicative of mixed inflammation with mild plasma cell hyperplasia. Bacterial and fungal cultures and PCR tests for *Sporothrix* and *Bartonella* all returned negative results. However, a *M. tuberculosis* complex rtPCR test, performed at the Real-time PCR Research and Diagnostics Core Facility, University of California–Davis was positive.

In the days following surgery, the patient’s clinical condition worsened significantly, and euthanasia was elected. The carcass was submitted for postmortem examination. The positive *M. tuberculosis* complex rtPCR result was returned later on the day of euthanasia and was not known at the time of submission for postmortem examination. Routine Containment Level-1 biosafety measures in accordance with the Canadian Biosafety Standard (Public Health Agency of Canada, https://www.canada.ca/en/public-health/services/canadian-biosafety-standards-guidelines/second-edition.html) were taken during the postmortem examination.

At gross examination, most lung lobes contained innumerable, 1–3-mm, firm, tan nodules throughout the parenchyma (Fig. 1). Within the cranial mediastinum, there were numerous, 1–3-cm nodules with homogeneous caseous tissue on cut surface. Within the dorsal mediastinum, near the heart base, were two, 3.5- and 7-cm diameter masses composed of a similar material (Fig. 2); the left cranial lung lobe had extensive fibrous adhesions to these masses. Several large caseous nodules were also present in the right middle, left cranial, and left caudal lung lobes (Fig. 3).

**Figures 1–3.**
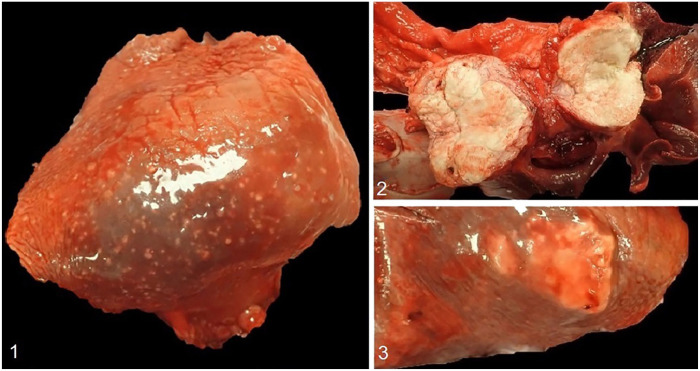
*Mycobacterium tuberculosis* infection in a dog. **Figure 1.** Caseous nodule in the left caudal lung lobe. **Figure 2.** Large, up to 7-cm diameter, caseous masses (cut surface) in the dorsal mediastinum, located in the region of the tracheobronchial lymph nodes at the base of the heart. **Figure 3.** Innumerable punctate granulomas distributed throughout the right caudal lung lobe.

All lobes of the liver contained caseous nodules of up to 1.5-cm diameter. In the left lateral and right medial liver lobes, 20–40% of the parenchyma was replaced by large (up to 10-cm diameter) masses of pale-yellow, friable, caseous material. Similar lesions were also noted throughout the omentum and kidneys. Sections of all main organs were placed in 10% neutral-buffered formalin, processed routinely, and stained with H&E for histologic examination.

Histologically, cumulatively throughout all examined lung sections, up to 20% of the pulmonary parenchyma was effaced by multifocal-to-coalescing granulomas. The granulomas had an almost pure population of macrophages with minimal accompanying neutrophils, lymphocytes, or plasma cells, and variably necrotic and/or mineralized centers. The thoracic lymph nodes were entirely necrotic with a thin rim of epithelioid macrophages (Fig. 4). Lesions in the liver, omentum, and kidney had a similar histologic appearance. Additionally, microscopic granulomas were noted in the pancreas and adrenal glands.

**Figure 4.**
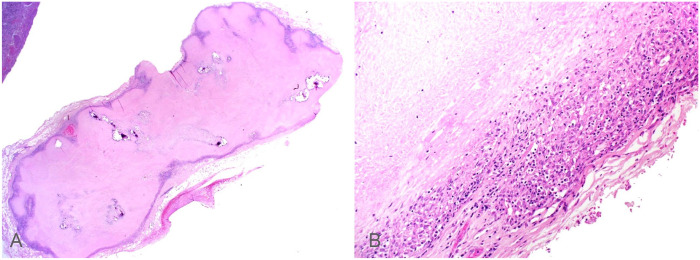
Granulomatous inflammation and necrosis in the mediastinal lymph node of a dog with *Mycobacterium tuberculosis* infection. H&E. **A.** Effacement of mediastinal lymph node architecture by necrosis, foci of mineralization, and granulomatous inflammation. Original objective 1.25×. **B.** Higher objective inspection reveals a thin rim of epithelioid macrophages with scattered lymphocytes that surrounds vast areas of necrosis. Original objective 20×.

Ziehl–Neelsen acid-fast stains performed on lung, liver, and omental tissues did not highlight any acid-fast microorganisms. Slides from the original mediastinal lymph node biopsy were then reviewed, and a single cluster of acid-fast bacilli was observed within a large field of necrosis with no immediately adjacent cell population. These organisms were strongly acid-fast in sections stained with Fite acid-fast and weakly acid-fast in sections stained with Ziehl–Neelsen stains. Lung, lymph node, liver, and kidney samples were subsequently submitted to both Public Health Ontario (PHO) and the Canadian Food Inspection Agency (CFIA) for mycobacterial culture and speciation.

Mycobacterial culture and susceptibility testing performed by PHO and CFIA confirmed the presence of a mycobacterial species that was susceptible to medications used in the treatment of tuberculosis in humans (isoniazid, rifampin, ethambutol, pyrazinamide). Initial analysis of this strain by means of an antigen detection assay (SD Bioline TB Ag MPT64; Abbott) confirmed that this isolate belonged to the *M. tuberculosis* complex.^4^ Further characterization within the *M. tuberculosis* complex was achieved by use of biochemical tests and line-probe assays (Bruker, Hain Lifescience), which confirmed the isolate as *M. tuberculosis*.^7^

Whole-genome sequencing (WGS) of the isolate was performed using an Illumina sequencing platform. The isolate was further characterized by classical 24-loci mycobacterial interspersed repetitive unit variable-number tandem repeats (MIRU-VNTR) and spoligotyping.^2,12^ The isolate’s MIRU-VNTR profile 2-3-4-3-4-3-3-1-2-2-4-4-2-2-5-1-6-3-3-3-3-11-2-2 was cross-referenced with available public health databases. Two identical genotypes were identified in Quebec, and 3 related genotypes were identified in Ontario. The Ontario cases were affiliated with foreign-born individuals with no epidemiologic links to the patient. One human *M. tuberculosis* isolate from Quebec was sequenced, and the WGS data comparison revealed that the canine isolate had a nearly identical genome sequence, suggesting a direct epidemiologic link. Provincial privacy laws precluded further investigation of the Quebec cases.

This animal was allocated as a high infectious risk based on the presence of acid-fast bacilli on smears of post-mortem lung samples, the extensive pulmonary involvement of disease, and the size of the dog, her lung capacity, and breathing patterns. The period of infectivity, defined as the potential time period during which a *M. tuberculosis*–infected individual may have been infectious to contacts, was estimated by public health authorities (based on established protocols used in human *M. tuberculosis* cases) to extend from 12 wk prior to onset of clinical signs until the animal’s euthanasia; a total of 14 mo. Tuberculin skin testing or interferon-gamma release assay (IGRA) serology was offered to all individuals who met the exposure criteria during this time (Table 1). Contact tracing and testing was coordinated by regional public health authorities (i.e., Wellington-Dufferin-Guelph Public Health, Toronto Public Health). Despite prolonged and close exposure of this dog to numerous household and veterinary personnel contacts during the estimated period of infectivity, testing of potentially exposed humans did not identify any clear evidence of canine-to-human transmission. All individuals who returned positive tuberculin tests had additional risk factors for previous sensitization to *M. tuberculosis* antigen (i.e., foreign-born, and previous BCG vaccination).

**Table 1.** Exposure criteria used to identify potential contacts and the outcomes of tuberculin skin testing (TST) or interferon-gamma release assay (IGRA) serology.

Contact type	Exposure criteria	Outcomes
Household contacts (2)	Everyone in household.	1. Negative initial and post 8-wk TST—Canadian-born 2. Negative post 8-wk IGRA—foreign-born
Close non-household contacts (2)	>96 h of exposure if ≥5 y/o, or >36 h exposure if immunosuppressed or <5 y/o.	1. Negative initial TST—declined post 8-wk TST given perceived low risk. 2. Declined all testing.
Veterinary clinics (3)	Any staff involved in direct care of the dog for ≥36 h of cumulative exposure. All staff involved in aerosolizing procedures (including intubation) unless the staff member wore personal protective equipment (fit-tested N95 mask) and/or any staff that sustained a contaminated needlestick injury during the treatment of the dog. All pathologists involved and all staff present during the postmortem examination.	Post 8-wk TST offered to these clients: Clinic 1: 53 staff met exposure criteria 31 tested 3 positives (all had other risk factors, i.e., foreign-born and BCG vaccination) Clinic 2: 6 staff met criteria—all negative TST Clinic 3: No staff met criteria

Diagnosis of mycobacterial infections in canine species has decreased throughout the course of the 20th and 21st centuries. Historically, *Mycobacterium bovis* was the agent that was isolated most commonly in cases of canine mycobacteriosis. The incidence of these infections has decreased with the advent of improved food safety standards and *M. bovis* eradication schemes. However, recent reports of endemic infection in African wild dogs and outbreaks among English foxhounds indicate that sporadic *M. bovis* infection still occurs.^3,8^

Infection of canids with *M. tuberculosis* occurs sporadically. Traditionally, humans are considered to be the reservoir for most canine infections, with many reported cases in dogs including a history of prolonged contact with an *M. tuberculosis*–infected human.^5,11^ The anthroponotic potential of *M. tuberculosis* carries implications for both human and animal health given that experimentally infected dogs are capable of infecting other animals kept in close contact, and naturally infected dogs with clinical disseminated mycobacteriosis can shed *M. tuberculosis* in various body fluids, including nasal secretions.^1,5^ As such, even though current evidence suggests that the zoonotic risk of canine *M. tuberculosis* only extends to veterinary professionals performing postmortem procedures, canine-to-human transmission should, nonetheless, still be treated as a potential event in any interaction with a *M. tuberculosis–*infected dog, and appropriate precautions should always be followed when handling the dog or its biological products.^10^ The potential for *M. tuberculosis* to move from human to canine populations is supported by the finding that 1 of 100 (1%) stray dogs living in a high-risk community developed clinical tuberculosis; 12 of 24 (50%) dogs living on a property with a sputum smear–positive human *M. tuberculosis* patient had evidence of immunologic sensitization to *M. tuberculosis*.^9^ This latter finding highlights the intriguing potential of *M. tuberculosis*–infected dogs to act as sentinels for unidentified infections in cohabiting human populations. Further, our case and similar studies suggest that household pets should be strongly considered for inclusion as part of the initial contact screening process in newly diagnosed cases of human *M. tuberculosis*.

Patient history prior to adoption was limited in our case, although it is known that this animal spent its early life in a remote community with historically high rates of active *M. tuberculosis* infections. Attempts at follow-up with the rescue group that removed the dog from that community and rehomed it in Ontario were unsuccessful.

As in other reported cases, definitive diagnosis was achieved late in the course of disease.^6^ Earlier detection could potentially have reduced animal suffering and owner expense, as well as advanced the public health response to the source of infection. However, numerous infectious agents may cause granulomatous inflammation in dogs and, in some regions, including southern Ontario, sterile and/or autoimmune granulomatous inflammation is anecdotally more common than infectious granulomatous inflammation. Because of financial considerations, it is unlikely in most cases that all potential organisms can be investigated. It is a regionally standard practice to perform acid-fast staining on tissues with granulomatous inflammation, as was done in our case. Our case emphasizes, however, that acid-fast staining on tissues may not be sufficiently sensitive. It is recommended to consider PCR testing for *Mycobacterium* spp. for any case that has been adopted from a region with a higher risk for tuberculosis, when other more common infectious agents have been ruled-out, and when an animal has failed to respond to immunosuppression. Our case clearly illustrates the importance of a thorough history in establishing a veterinary patient as a potential zoonotic risk. The veterinarians managing our case were not aware that human tuberculosis was endemic in the region from which this dog was procured, and PCR testing for mycobacterial pathogens was performed along with testing for other infectious and non-infectious diseases to address all differential diagnoses being considered. Were the details of animal origin known from the outset, animal suffering, owner expense, and human exposure could likely have been greatly reduced by advanced testing performed early in the course of disease.

The delay in obtaining a definitive diagnosis in our case until late in the clinical course was no doubt contributed to by the sparsity of readily available antemortem and minimally invasive mycobacterial tests. The utility of IGRA serology, a validated method used for the diagnosis of mycobacteriosis in humans and cattle, has been applied only sporadically in the diagnosis of mycobacteriosis in canids.^8,9^ An obvious advantage of IGRA serology is the need for only a single blood sample to establish evidence of immunologic sensitization to mycobacterial proteins. The use of IGRA serology in dogs remains confined to the use of novel techniques in experimental settings that remain to be validated and offered commercially. Although IGRA serology performed early in the disease course in our patient may have indeed minimized animal suffering, owner expense, and public health repercussions, to our knowledge, commercial or logistically viable testing options were not available in North America at the time of this animal’s hospitalization. Given the apparent trend in veterinary literature to encounter significant delays in the diagnosis of canine *M. tuberculosis* infections, it seems prudent for efforts to be made in developing a validated canine IGRA to be available readily, and for animal health practitioners to be made aware of the existence and utility of such tests. In the absence of a readily available canine IGRA, and given the potential public health concerns of undiagnosed mycobacterial infections, we recommend that animal health practitioners consider pursuing molecular testing, such as *Mycobacterium* spp. rtPCR, early in the disease course for canine patients with chronic granulomatous disease, rather than relying on tests that are sub-optimally sensitive for the diagnosis of paucibacillary mycobacterial infections (i.e., acid-fast stains). If molecular tests are not to be opted for from the outset of the diagnostic investigation, we recommend that fresh (i.e., non–formalin-fixed) tissues be frozen at the time of biopsy sampling so that further testing can be pursued in the event of non-diagnostic or inconclusive histopathology results.

We report here the diagnostic and public health investigation of a *M. tuberculosis* infection in a dog within Canada. We also highlight the importance of interdepartmental communication when treating veterinary patients infected with potentially zoonotic agents. The number of individuals put at risk of exposure was likely increased by lack of timely warnings to adjunctive staff involved in case management. The anesthesiology service tasked with preparing this patient for surgery were not adequately alerted to the possibility of respiratory zoonoses prior to cough-inducing procedures (i.e., intubation). Similarly, the attending clinicians were not aware of the *M. tuberculosis* complex–positive PCR result until after the body was sent for postmortem examination, and the duty pathologists were not alerted. Management of our case served to reaffirm to clinicians and adjunctive staff at OVC the potential of zoonotic, albeit rare, etiologies of granulomatous disease in dogs. Additionally, our case prompted a review of protocols relating to the postmortem management of animals with potentially zoonotic disease. Specifically, routine cleaning procedures were changed to minimize the aerosolization of biological matter (e.g., prohibiting spraying of table surfaces with high-pressure water sources). Additionally, postmortem examination of granulomatous disease in dogs is now advised to be performed in a dedicated isolation unit in which more stringent personal protective measures are required (i.e., N95 facemasks, or powered air-purifying respirators), and a logging system for all personnel entering the isolation unit has been introduced.
